# Aging affects gain and internal noise in the visual system

**DOI:** 10.1038/s41598-020-63053-0

**Published:** 2020-04-21

**Authors:** Fang-Fang Yan, Fang Hou, Hongyu Lu, Jia Yang, Lijun Chen, Yifan Wu, Ge Chen, Chang-Bing Huang

**Affiliations:** 1 Key Laboratory of Behavioral Science, Institute of Psychology, Chinese Academy of Sciences, Beijing, 100101 China; 2 Department of Psychology, University of Chinese Academy of Sciences, Beijing, 100049 China; 3 School of Ophthalmology & Optometry and Eye Hospital, Wenzhou Medical University, Wenzhou, 325027 China; 4 Maternal and Child Health Care Hospital of Qinhuangdao, Qinhuangdao, 066000 China; 5 School of Art and Design, Zhengzhou University of Light Industry, Zhengzhou, 450000 China

**Keywords:** Sensory processing, Human behaviour

## Abstract

Visual functions decline with age, but how aging degrades visual functions remains controversial. In the current study, the mechanisms underlying age-related visual declines were examined psychophysically. We developed an efficient method to quickly explore contrast sensitivity as a function of nine spatial frequencies at three levels of external noise in both young and old subjects. Fifty-two young and twenty-six old subjects have been screened for ophthalmological and mental diseases and participated in the experiment. Contrast sensitivity varied significantly with spatial frequency, age, and level of external noise. By adopting a nonlinear observer model, we decomposed contrast sensitivity into inefficiencies in internal additive noise, internal multiplicative noise, perceptual template gain, and/or system non-linearity. Model analysis revealed that aging impacts both internal additive noise and perceptual template gain, and such age-related degradation is tuned to spatial frequency, which is also a good predictor to discriminate old from young. The quick characterization of contrast sensitivity functions at different noise levels and the accompanying analysis developed in the current study may have profound application in other clinical populations.

## Introduction

Aging is an unavoidable process in human life and affects almost all sensory and cognitive functions. As far as vision is concerned, aging impacts visual acuity^1^, contrast sensitivity^2–4^, motion^5,6^, contour perception^7,8^, and the useful field of view^9,10^, and so on. In the current study, we focused on how aging affects contrast sensitivity, one of the most fundamental visual functions^11^.

Contrast sensitivity, depicting creatures’ ability to detect luminance difference, is critical for daily vision. Nameda, *et al*.^12^ found that contrast sensitivity at high spatial frequencies started to decrease from 40 years old and decreased at all frequencies from 50–60 years old. Owsley, *et al*.^13^ showed that contrast sensitivity began to fall at medium and high spatial frequencies after the age of 40 to 50 years. In a later study, Sloane, *et al*.^14^ reported that contrast sensitivity decreased at all spatial frequencies and the decline was particularly prominent at high spatial frequencies. Although there were some discrepancies in the observed contrast sensitivity deficits in old observers, the declining pattern was largely consistent among published studies, i.e. contrast sensitivity began to decrease from high spatial frequencies and then extended to all frequencies after about 60 years old.

On the other hand, how aging affects contrast sensitivity remains controversial. Based on the noise titration method and linear amplification model (LAM)^15,16^, previous psychophysical studies attributed the decline of contrast sensitivity in the elderly to sampling inefficiency and/or internal noise elevation. Pardhan, *et al*.^17^ reported that the loss of contrast sensitivity to grating of 6 cycles per degree (c/d) in aged observers resulted from lowered calculation efficiency without any significant increase of internal noise; Bennett, *et al*.^18^ tested contrast sensitivity at the spatial frequencies of 1, 3, and 9 c/d, and found that only calculation efficiency reduced in aged observers. In another study, Pardhan^19^ claimed that lower calculation efficiency contributed to the declines of contrast sensitivity at 1 and 4 c/d, whereas elevated equivalent noise played a more important role at spatial frequency of 10 c/d in old subjects, which implied that different mechanism(s) were involved in the aging process. Contrary to previous studies that used local and static external noise, Allard, *et al*.^20^ adopted extended dynamic noise and concluded that age-related contrast sensitivity loss resulted from elevated internal equivalent noise at low spatial frequency (1c/d) and decreased calculation efficiency (probably combined with increased internal equivalent noise) at higher spatial frequencies (3 and 9 c/d). Arena, *et al*.^21^ investigated the sensitivity to motion direction and orientation in young and old adults and demonstrated that aging affected only the internal noise but not the calculation efficiency at low spatial frequency (1 c/d). The inconsistent results among these studies may be due to different spatial-temporal properties of the external noise patterns, the distinct stimulus attributes (e.g. spatial frequency), presentation procedures and/or the availability of feedback.

It is important to point out that almost all previous studies were adhered to the LAM model, which posited that visual signal was analyzed in a linear fashion and the only noise source was additive noise, i.e. irrespective of the input stimulus^15^. It’s now widely accepted that our visual system is intrinsically non-linear and/or hosts different noise sources^22–26^. Observer model that incorporates nonlinear transducer function and internal  multiplicative noise, i.e. perceptual template model (PTM), has been found to provide better account for visual deficits in amblyopia^27^ and dyslexia^28^, system changes after perceptual learning^29–31^ and playing video game^32^, and attention modulation^33,34^. In the current study, we attempted to re-examine the intrinsic psychophysical mechanisms underlying age-related change(s) of contrast sensitivity at a variety of spatial frequencies in a relatively large cohort of young and aging populations. With the development of a quick characterization method of contrast sensitivity functions at multiple external noise levels and the application of a non-linear observer model analysis, we found that aging significantly lowered perceptual template gain and elevated internal additive noise in a frequency-dependent fashion.

## Results

### Contrast sensitivity function (CSF)

**Figure 1:**
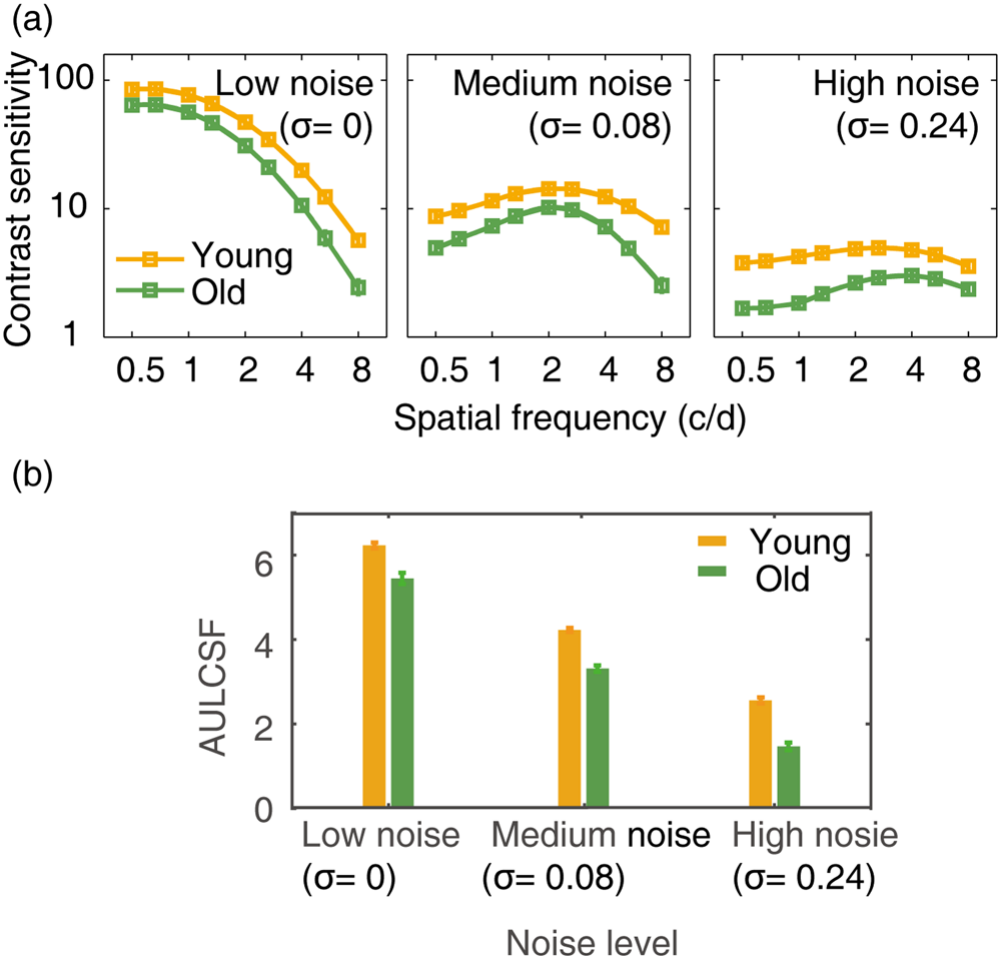
(**a**) Contrast sensitivity functions at three external noise levels for the average observer of the young (orange) and old (green) group, respectively. (**b**) Schematic diagram of the AULCSF at low, medium and high noise levels. The orange and green bars denote the young and old group, respectively. Error bar represents standard error.

The CSFs at three external noise levels were presented in Figure 1a. An analysis of variance (ANOVA) showed that contrast sensitivity varied significantly with spatial frequency (*F*(8, 608) = 18.095, *p* = 2.247 × 10^−24^), external noise level (*F*(2, 152) = 140.082, *p* = 3.242 × 10^−35^), and age (*F*(1, 76) = 157.289, *p* = 3.420 × 10^−20^). There were significant interactions between spatial frequency and age (*F*(8, 608) = 4.199, *p* = 6.535×10^-5^), and between spatial frequency and noise level (*F*(16, 1216) = 46.337, *p* = 1.449 × 10^−113^), but not between noise level and age (*F*(2, 152) = 1.549, *p* = 0.216). The interaction among spatial frequency, noise level, and age was also significant (*F*(16,1216) = 23.933, *p* = 1.542 × 10^−61^). Our results indicated that age-related decline in contrast sensitivity was frequency-dependent, consistent with previous studies^13,14,19^.

To index the difference in contrast sensitivity between young and old subjects, we derived the Area Under the Log CSF (AULCSF) for the two groups (Figure 1b). An ANOVA showed that AULCSF differed significantly with external noise level (*F*(2, 152) = 131.286, *p* = 7.629 × 10^−34^) and age (F(1, 76) = 140.003, *p* = 6.498 × 10^−19^), but not the interaction between external noise level and age (*F*(2, 152) = 2.247, *p* = 0.109). Averaged across observers, the AULCSF of old group was 87%, 78%, and 57% of that in the young group at low, medium and high noise levels, respectively.

### Mechanism of contrast sensitivity declines

**Figure 2:**
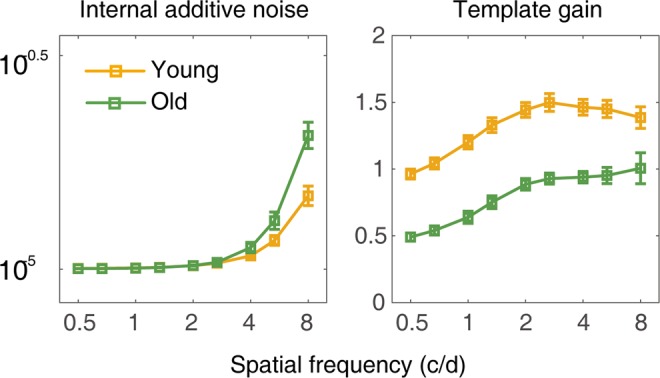
The estimated PTM parameters (left: internal additive noise; right: template gain) at nine spatial frequencies averaged for the young (orange) and old (green) groups, respectively.

We fitted PTM to the CSFs for each observer in young and old groups. Averaged across subjects, the model accounted for 91.67% (±1.00%, s.e.) and 88.09% (±1.00%) of data variance in young and old groups, respectively. Although internal multiplicative noise and system non-linearity varied across subjects, they were invariant with spatial frequencies^35,36^. The internal multiplicative noise was 0.155 ± 0.017 and the system non-linearity was 1.459 ± 0.030 in the old group, and 0.158 ± 0.010 and 1.523 ± 0.027 in the young group, respectively. There was no significant difference in internal multiplicative noise (*t*(76) = 0.206, *p* = 0.837) and system non-linearity (*t*(76) = 1.489, *p* = 0.141) between the two groups. Average internal additive noise and template gain at the nine spatial frequencies were showed in Figure 2. An ANOVA revealed that internal additive noise varied significantly with age (*F*(1, 76) = 9.620, *p* = 0.003) and the interaction of spatial frequency and age (*F*(8, 608) = 11.086, *p* = 1.110 × 10^−14^), but not spatial frequency (*F*(8, 608) = 0.229, *p* = 0.986). Post-hoc comparison revealed that internal additive noise was significantly higher (by 75.58% on average) in the aged group than that in the young group at the two highest spatial frequencies, i.e. 5.33 and 8 c/d (*t*(76) = −2.143, *p* = 0.035; *t*(76) = −3.636, *p* = 0.001, respectively). Our results indicated that aging impacted internal additive noise mostly around high frequency domain.

Template gain varied significantly with spatial frequency (*F*(8, 608) = 6.153, *p* = 1.196 × 10^−7^) and age (*F*(1, 76) = 53.426, *p* = 2.284 × 10^−10^) but not the interaction of the two factors (*F*(8, 608) = 1.033, *p* = 0.410), indicating an overall down-regulation of system gain. The magnitude of gain significantly lowered by 39.44% in the aged group averaged across all spatial frequencies.

### Correlation among different measures

#### Contrast sensitivity & internal additive noise

**Figure 3:**
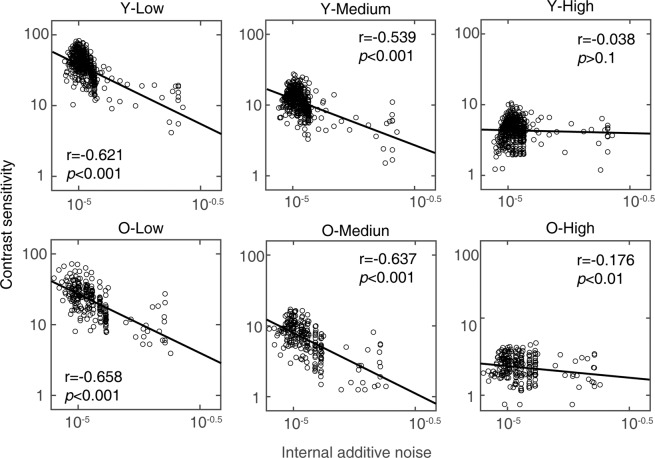
The correlations between contrast sensitivity and internal additive noise at all spatial frequencies at the low, medium and high noise levels for young and old groups, respectively. Data from all observers were pooled. ‘Y’ denotes the young group; ‘O’ denotes the old group; ‘Low’ denotes the condition of the low noise level; ‘Medium’ denotes the condition of the medium noise level; ‘High’ denotes the condition of the high noise level. The data were the residual plus mean values after excluding the effect of spatial frequency.

We employed partial correlation analysis (two-tailed) to probe the potential relationships between contrast sensitivity and internal additive noise while excluding the effect of spatial frequency. In addition, the percentile bootstrapping (2,000 bootstrap samples) was used to construct 95% confidence interval (CI) to assess the statistical significance of these correlations. The results showed that contrast sensitivity significantly correlated with internal additive noise at all three external noise levels for the old group (low noise: *r* = −0.658, *p* = 2.497 × 10^−30^, 95% bootstrap CI = [−0.746, −0.556]; medium noise: *r* = −0.637, *p* = 6.765 × 10^−28^, 95% bootstrap CI = [−0.718, −0.545]; high noise: *r* = −0.176, *p* = 0.007, 95% bootstrap CI = [−0.311, −0.046]); and in the low (*r* = −0.621, *p* = 4.014 × 10^−51^, 95% bootstrap CI = [−0.690, −0.542]) and medium (*r *= −0.539, *p* = 1.372 × 10^−36^, 95% bootstrap CI = [−0.634, −0.428]) but not high noise (*r* = −0.038, *p* = 0.409, 95% bootstrap CI = [−0.151, 0.066]) levels for the young group. Figure 3 showed the residual plus mean values of contrast sensitivity and internal additive noise at nine spatial frequencies after excluding the effect of spatial frequency.

#### Contrast sensitivity & system template gain

**Figure 4:**
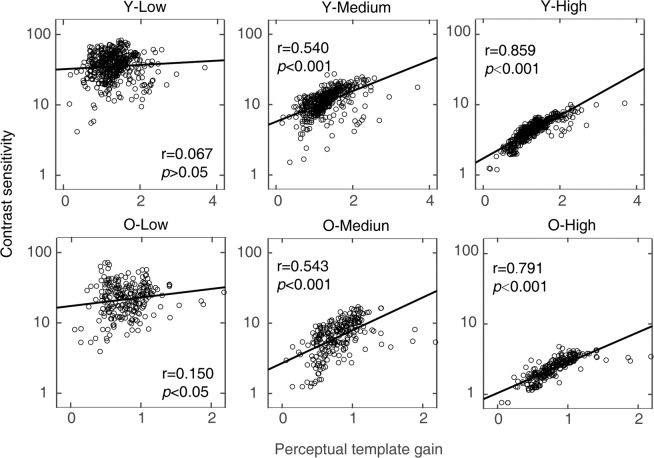
The correlations between contrast sensitivity and the gain of perceptual template at all spatial frequencies at low, medium and high noise levels for the young and old group, respectively. Data from all observers were pooled. ‘Y’ denotes the young group; ‘O’ denotes the old group; ‘Low’: low noise level; ‘Medium’: medium noise level; ‘High’: high noise level.

Partial correlation analysis showed that contrast sensitivity (after excluding the effect of spatial frequency) positively correlated with template gain at medium and high external noise levels (*r* = 0.540, *p* = 1.223 × 10^−36^, 95% bootstrap confidence interval = [0.453, 0.626]; *r* = 0.859, *p* = 4.751 × 10^−137^, 95% bootstrap confidence interval = [0.830, 0.886], respectively), but not at the low noise level (*r* = 0.067, *p* = 0.146, 95% bootstrap confidence interval = [−0.041, 0.174]) for the young group, and at all three noise levels (low noise: *r *= 0.150, *p* = 0.022, 95% bootstrap confidence interval = [0.018, 0.281]; medium noise: *r* = 0.543, *p* = 2.989 × 10^−19^, 95% bootstrap confidence interval = [0.435, 0.653]; high noise: *r* = 0.791, *p* = 3.8040 × 10^−51^, 95% bootstrap confidence interval = [0.734, 0.857]) for the old group (Figure 4).

#### Visual acuity & CSF

We conducted Pearson correlation analysis (two-tailed) on visual acuity (logMAR) and AULCSF in the young and old groups. The results showed that visual acuity correlated significantly with AULCSF at low noise level (*r* = −0.274, *p* = 0.049) but not at medium (*r* = −0.106, *p* = 0.456) and high noise levels (r = −0.105, *p* = 0.460) in the young group. On the contrary, visual acuity correlated significantly with AULCSF at the high noise level (*r *= −0.482, *p* = 0.013), but not at the low (*r* = −0.276, *p* = 0.172) and medium noise levels (*r* = −0.349, *p* = 0.081) in the old group.

### Classification between old and young

#### CSF and visual acuity as predictors

The remarkable difference of CSFs between the old and young groups prompted us to evaluate whether CSF is a good indicator of aging. We selected AULCSF, cutoff spatial frequency (cutSF) that characterizes the high spatial frequency limit of spatial vision and visual acuity as predictors. We used a logistic regression analysis to predict membership in the two groups:

1 $$P(old|AULCSF,cutSF,\,visual\,acuity)=\frac{\exp ({\theta }_{0}+{\theta }_{i,m}\cdot AULCS{F}_{m}+{\theta }_{j,m}\cdot cutS{F}_{m}+{\theta }_{z}\cdot visual\,acuity)}{1+\exp ({\theta }_{0}+{\theta }_{i,m}\cdot AULCS{F}_{m}+{\theta }_{j,m}\cdot cutS{F}_{m}+{\theta }_{z}\cdot visual\,acuity)}$$

where *P*(old) is the probability of a particular subject being recognized as an old observer, *θ*_0_ indexes the constant, *θ*_*i,m*_ indexes the coefficient corresponding to AULCSF, *θ*_*j,m*_ indexes the coefficient corresponding to cutSF, θz indexes the coefficient corresponding to visual acuity, and *m* indexes the noise level.

The prediction analysis revealed two predictors, i.e. AULCSF and cutSF at the middle noise level, leaded to an accuracy up to 94.9% (Hosmer and Lemeshow goodness-of fit, χ^2^(8) =13.991, *p* = 0.082). The coefficients of the two predictors and *θ*_0_ were −7.613 (*p* = 0.013), −2.313 (*p* = 0.001), and 37.597 (*p* = 0.001), respectively. AULCSF itself discriminated membership of age at a rate of 91.0% (Hosmer and Lemeshow goodness-of fit test, χ^2^(8) = 4.2423, *p* = 0.817). The coefficients of the predictor and *θ*_0_ were −7.179 (*p* = 6.267 × 10^−5^) and 26.392 (*p* = 8.220 × 10^−5^), respectively. In other words, AULCSF and cutSF at the middle noise level were the critical factors for discriminating the aged from the young. Moreover, the AULCSF at the middle noise level made a greater contribution tothe discrimination.

#### PTM parameters as predictors

The PTM characterized contrast sensitivity with internal additive noise, template gain, internal multiplicative noise, and non-linearity. We fed the four fitted parameters as predictors into a logistic regression model (similar to Eq. 1) to evaluate whether the PTM was a good indicator of age. Internal additive noise and gain were classified as the best predictors (Hosmer and Lemeshow goodness-of fit-test, χ^2^(8) =1.420, *p* = 0.994), with an accuracy of 93.60%. The coefficients of the two factors and *β*_0_ were 140.562 (*p* = 0.002), −13.641 (*p* = 4.225 × 10^−4^), and 8.94 (*p* = 0.001), respectively.

## Discussion

Aging profoundly degrades contrast sensitivity and the magnitude of declines depends on spatial frequency. Our results also suggest that the increase of internal additive noise at high spatial frequencies and the decrease of template gain over a wide range of spatial frequencies underlie the age-related degradation in contrast sensitivity.

Our results indicated different aging mechanisms of contrast sensitivity. At low to medium spatial frequency, aging affected only perceptual template gain but not internal additive noise; at high frequencies, aging impacted both template gain and internal additive noise. The finding of frequency-dependent increase of internal additive noise was similar with Pardhan^19^ who found the internal additive noise played an important role on contrast sensitivity at high frequency (10 c/d) but not lower frequencies (1, 4 c/d), but contrary to Pardhan, *et al*.^17^, Bennett, *et al*.^18^, Allard, *et al*.^20^, and Silvestre, *et al*.^37^. At one hand, many optical^38–40^ and neural factors^41–43^ contributed to the internal noise in visual system and internal additive noise itself may have different components (e.g. photon noise, early and late noise in Silvestre *et al*.,^37^). For example, smaller pupil size, lower optical density of the ocular media, and decreased light absorption in the aged population, may have strong influence over contrast sensitivity at high spatial frequency (Owsley, 2011), albeit the optical contribution was minimized through ophthalmological examinations. On the other hand, the characteristic of subjects (e.g. screening criterion), testing frequency (e.g. low vs high), and selected theoretical model (e.g. linear vs non-linear; single noise vs multiple noises) may also perplex the interpretation of age-related declines in contrast sensitivity. For example, subjects were included according to self-report of no known ocular diseases in the study of Bennett, Sekuler, and Ozin (1999). We performed a set of thorough ophthalmological examinations for all our subjects to minimize potential optical influence on contrast sensitivity. In addition, linear model has been reported to have difficulties in explaining several findings that related to processing inefficiency or changes of visual system^44^.

The down-regulation of GABAnergic inhibition in visual cortex may be the neural cause for internal additive noise elevation in old population. Leventhal, *et al*.^42^ found that the exertion of GABA or its receptor agonist rejuvenated V1 neurons of aged monkeys, exhibiting enhanced intracortical inhibition, increased signal-to-noise ratios, and reduced excitability, which suggest that GABA declines may be the basis of abnormal response properties of aging cells. Using Nissl staining and immunohistochemical techniques, Hua, *et al*.^43^ found that the density of GABA-immunoreactive neurons and the ratio of GABA-immunoreactive neurons to total V1 neurons of aged cats were significantly lower than those of young cats, which provided direct morphological evidence of decreased GABAergic inhibition in the striate visual cortex of senile animals. In human studies, with the help of magnetic resonance spectroscopy (MRS), researchers have provided some preliminary evidences that occipital GABA was significantly lower in old observers compared to the young observers^45,46^, and positively associated with task performance^45^.

The perceptual template or filters describe the observer’s overall tuning characteristics for a signal-valued stimulus, e.g., a spatial frequency filter with a center frequency of the highest gain and a bandwidth such that a range of frequencies adjacent to the center frequency passing through with smaller gains^22,33^. In this study, we found that aging impacted perceptual template gains over all tested spatial frequencies.

For the young group, contrast sensitivity at low noise level was negatively correlated with internal additive noise (but not perceptual template gain) and contrast sensitivity at high noise level was positively correlated with the perceptual template gain (but not internal additive noise), consistent with our previous study^36^. But in the old group, contrast sensitivity was negatively correlated with internal additive noise and positively correlated with the perceptual template gain at all the noise levels. We further compared the correlation strength between different measures in the two groups with Fisher z-transformation. The results indicated that the correlations between internal additive noise and contrast sensitivity of old group were significantly greater than that of young group at medium (*p* = 0.031, single sided test) and high noise (p = 0.041) levels; and the correlation between perceptual template gain and contrast sensitivity in the old group was significantly smaller than that in the young group at high noise level (p = 0.004). We argued that different information processing mechanisms may be involved in young subjects when exposed to different levels of external noise, and aging changed the processing mechanisms.

In this study, we used external noise titration method to quantify the psychophysical mechanism(s) underlying age-related declines in contrast sensitivity. This method and its associated noise settings have been widely used in earlier studies^27–34^. Allard and his colleagues proposed that the property (e.g. intensity and/or spatiotemporal characteristics) of external noise might affect subject’s information processing strategy and thus change the interpretation of some visual phenomena^20,37,47–49^. For example, with spatiotemporal extended noise, Allard, *et al*. (2013) found that aging lowered calculation efficiency at high spatial frequency, a finding similar to previous studies^17,18^, but only elevated internal additive noise at low spatial frequency, different from early studies^18,19^. With spatially local and temporally extended external noise, we found that aging impacted both internal additive noise and template gain across all tested frequencies, although the effects varied with spatial frequency. Since the characteristic of subjects, the way of how noise and signal integrated (spatial vs temporal), and the assumption of visual information processing (e.g. linear vs non-linear), it’s unlikely to commit to any of the conclusions presently and new studies to directly compare all relevant experimental setups and theoretical frameworks are needed in the future.

It’s worthy to note that we strictly screened our subjects for ophthalmological diseases and other diseases that may affect task fulfillment. Despite the difference of visual acuity was remarkable (*t*(76) = −4.752, *p* = 9.310 × 10^−6^) between the young and old groups, the correlation between visual acuity and AULCSF was inconsistent in the young and old groups at three noise levels, which was similar to the result of Yan, *et al*.^35^ at the low (zero) noise level. These results indicated that the declines in contrast sensitivity in the aged group cannot be accounted by the degradation of visual acuity. In this regard, our results may better reflect normal or physiological age-related contrast sensitivity deficits. We concluded that aging elevated internal additive noise at high spatial frequencies and weakened template gain over all spatial frequencies, leading to a reduction of contrast sensitivity across a wide range of spatial frequencies.

## Methods

### Observers

Fifty-two young, aged 18 to 29 years (22.6 ± 2.6 yrs, mean ± s.d.), and twenty-six old subjects, aged 60 to 80 years (67.3 ± 6.1 yrs), participated in the experiment. The observers were recruited from nearby universities and/or communities (education> 9 yrs) and examined by an ophthalmologist (the third author). The inclusion criteria were: normal or corrected-to-normal visual acuity in both eyes (≥20/25), normal ocular media, free of retinal disease (e.g., strabismus, glaucoma, cataract, and macular degeneration), normal trichromatic vision, normal stereo vision, and free from history of mental diseases and cognitive deficits (MMSE score> 24). The average visual acuity was −0.201 ± 0.073 (logMAR, mean ± s.d.) in the young group and −0.117 ± 0.071 in the old group. Written informed consent was obtained from each observer after the nature of the study was explained. The experimental protocol was approved by the ethics review committee of Institute of Psychology, Chinese Academy of Sciences and all research activities were adhered to the tenets of the Declaration of Helsinki.

### Apparatus and stimuli

Stimuli were controlled by a computer running Matlab and PsychToolBox extensions^50,51^ and presented on DELL color monitor with a spatial resolution of 1600 × 1200 pixels, a refresh rate of 85 Hz, a mean luminance of 40.4 cd/m^2^, and a circuit-enabled and Gamma-corrected 14-bit gray resolution^52^. A chin rest was used to minimize head movements during the experiment. The observers viewed the display binocularly in a dimly lit room at a distance of 1.14 meters.

Stimuli were oriented gratings (45° or 135°) of nine spatial frequencies (0.5, 0.67, 1, 1.33, 2, 2.67, 4, 5.33, 8 c/d) at three levels of external noise (white noise, μ =0 and σ ∈ [0 0.08 0.24]). The size of signal gratings was inversely proportional to its frequencies, i.e., 6°, 4.5°, 3°, 2.25°, 1.5°, 1.125°, 0.75°, 0.563°, 0.375°, respectively. They had constant cycles (*N* = 3). A truncated-Gaussian envelope, whose size was 1:6 to the gratings, blended the image into background. External noise, with same size as the signal, was generated randomly in each trial and the size of noise elements also varied with spatial frequency of the masked grating such that the number of noise elements in each grating cycle was same across all spatial frequencies^36,53,54^.

### Procedure

**Figure 5:**
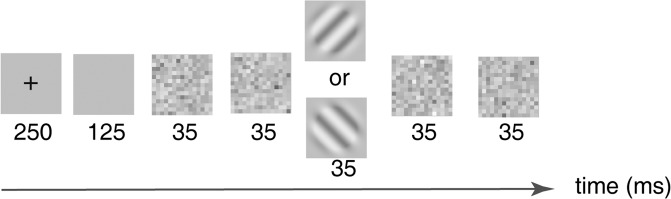
The trial procedure. Each trial started with a 250-ms fixation, followed by a 125-ms blank screen, two external noise frames, a grating or blank frame, and two additive external noise frames. Each frame lasted 35-ms.

A grating orientation identification task was used in the experiment (Figure5). Each trial started with a 250-ms fixation cross in the screen center with a brief tone signaling each trial’s onset, which was followed by 125-ms blank screen, two 35-ms frames of external noises, one 35-ms frame of signal grating with random orientation (45° or 135°), and two 35-ms additive frames of external noises. Observers reported the grating orientation with keyboard press. No feedback was provided. Inter-trial interval was 500 ms.

### Design

A quick procedure was used to evaluate CSFs at different noise levels^35,55^. Briefly, the quick method characterized CSF with a four-parameter log-parabola function, e.g. peak gain (or maximum contrast sensitivity), spatial frequency that corresponds to peak gain, low-frequency truncation, and bandwidth that defines the parabolic function’s full-width at half-height (in octaves). Different combinations of parameter values were assigned a prior as a four-dimensional probability density function (i.e. parameter space). The stimuli space consisted of the possible grating contrast ranged from 0.1% to 100% in 1 dB steps and nine spatial frequencies (0.5, 0.67, 1, 1.33, 2, 2.67, 4, 5.33, and 8 c/d). Based on observer’s response (correct or incorrect) on a grating of a certain spatial frequency and contrast, the parameter probability density function was updated using Bayes’ rule (i.e. posterior). The contrast and spatial frequency of the signal grating in next trial was chosen such that the expected entropy of the probability density function after that trial was the lowest (i.e. maximal information gain). The experiment consisted of 300 trials, with 100 trials per external noise level.

### Analysis

To obtain CSF at each noise level, we sampled 1,000 sets of CSF parameters from the posterior distribution of CSF parameters, constructed 1,000 corresponding CSF curves, and obtained the empirical distribution of the CSF. This re-sampling procedure automatically takes into account the covariance structure in the posterior distribution of the CSF parameters and allows us to assess the precision of the estimated CSF curve^35,56^.

The AULCSF, a summary metric of the CSF function^35,55,57,58^, was calculated as the integration of the area under the log CSF curve from 0.5 to 8 c/d. The cutSF was defined as the spatial frequency at which contrast sensitivity is 1.0.

**Figure 6:**
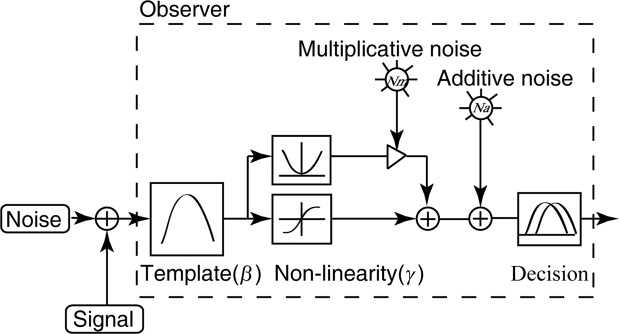
Flowchart of the perceptual template model (PTM; Lu & Dosher, 1999). The PTM involves five main components: a perceptual template, a nonlinear transducer, a multiplicative internal noise source, an additive internal noise source, and a decision process.

The perceptual template model (PTM, Figure6; Lu & Dosher, 1999) described an observer’s perceptual sensitivity as a function of performance level (*d’*) and external noise level with four free parameters, i.e. the signal non-linearity (*γ*), gain of the perceptual template (*β*) that expressed relative to the external noise, the internal additive noise whose intensity was invariant to input stimulus energy (*N*_*a*_), and internal multiplicative noise whose intensity co-varied with the input stimulus energy (*N*_*m*_):

2 $$c={\left[\frac{(1+{N}_{m}^{2}){N}_{ext}^{2\gamma }+{N}_{a}^{2}}{{\beta }^{2\gamma }\left(\frac{1}{{d}^{\text{'}2}},-,{N}_{m}^{2}\right)}\right]}^{1/2r}$$

where *c* is contrast threshold, *N*_*ext*_ is the external noise, *d’* is the performance level (80.3% percent correct for qCSF method or d’ = 1.705), and *γ*, *β*, *N*_*m*_, *N*_*a*_ are the four free parameters used to characterize system inefficiency.

The PTM model was fitted directly to the estimated CSF for each observer in the young and old groups by a weighted least-square method, which was used to minimize ${\sum }^{}{(C{S}_{predicted}-C{S}_{measured})}^{2}$. *CS*_*predicted*_ is the model-predicted contrast sensitivity (i.e. log(1/contrast threshold)) at the tested spatial frequencies and noise levels, and *CS*_*measured*_ is the measured contrast sensitivity. Our recent studies have demonstrated that the slope of contrast psychometric function was invariant to spatial frequencies and external noises^35,36^, which rules out the possibility that non-linearity (*γ*) and multiplicative noise (*N*_*m*_) change along with spatial frequency and external noise^22^. The goodness-of-fit was evaluated by *r*^2^:

3 $${r}^{2}=1.0-\frac{{\sum }^{}{(C{S}_{predicted}-C{S}_{measured})}^{2}}{{\sum }^{}{[C{S}_{measured}-mean(C{S}_{measured})]}^{2}}$$

## Data Availability

The data that support the findings of this study are available from the corresponding author upon reasonable request.
